# Metabolic Modulation by Dimethyl Fumarate Alters Docetaxel Responses in Prostate Cancer Cells

**DOI:** 10.3390/ijms27146209

**Published:** 2026-07-11

**Authors:** Andrés Coneo-Pretelt, Ana Peñata-Taborda, Lyda Espitia-Pérez, Luisa Jiménez-Vidal, Mario Negrette-Guzmán, Nathalia Jones-Cifuentes, Bladimiro Rincón-Orozco, Fabio Aristizábal-Gutiérrez, Pedro Espitia-Pérez

**Affiliations:** 1 Grupo de Investigación Biomédica y Biología Molecular, Facultad de Ciencias de la Salud, Universidad del Sinú, Montería 230001, Colombia; aconeo79@gmail.com (A.C.-P.); investigador01gibm@unisinu.edu.co (A.P.-T.); lydaespitia@unisinu.edu.co (L.E.-P.); lufejivi@gmail.com (L.J.-V.); 2 Departamento de Ciencias Básicas, Escuela de Medicina, Universidad Industrial de Santander, Bucaramanga 680002, Colombia; maneguz@uis.edu.co (M.N.-G.); nathalia.jones.cifuentes@gmail.com (N.J.-C.); blrincon@uis.edu.co (B.R.-O.); 3 Grupo de Investigación en Farmacología y Metabolismo (FARMET), Universidad Industrial de Santander, Bucaramanga 680001, Colombia; 4 Departamento de Farmacia, Facultad de Ciencias, Universidad Nacional de Colombia, Bogotá 111321, Colombia; faaristizabalg@unal.edu.co

**Keywords:** dimethyl fumarate, docetaxel, repurposing, combination therapy, metabolic reprogramming

## Abstract

Dimethyl fumarate (DMF) is a clinically approved fumarate ester with pleiotropic effects and a promising candidate for drug repurposing in cancer. Here, we investigated whether low-dose DMF could modulate docetaxel (DCT) responses in prostate cancer cells. PC-3, LNCaP, and RWPE-1 cells were exposed to DMF and DCT individually or in combination, and cell viability, drug interaction profiles, apoptosis, oxidative stress, mitochondrial mass, glutathione status, glucose consumption, lactate production, *LDHA/SOD2* expression, and oxygen consumption were evaluated. Low-dose DMF and DCT were well-tolerated in RWPE-1 normal prostate cells. In prostate cancer cells, the DMF–DCT combination was cytotoxic and strongly dose- and ratio-dependent, with the most favorable responses at higher exposures and under balanced or DCT-enriched regimens. DMF-DCT-treated LNCaP cells showed reduced viability, decreased lactate production, increased glucose consumption, mitochondrial dysfunction, oxidative stress, downregulation of *LDHA* and *SOD2*, and caspase-associated apoptosis. In contrast, PC-3 cells showed greater combinatorial susceptibility, a docetaxel dose-sparing effect, and a low glycolytic profile, with concomitant cytotoxicity and mitochondrial dysfunction. These findings identify DMF as a context-dependent metabolic modulator of docetaxel response, supporting further evaluation of DMF–DCT combinations as a potential therapeutic strategy in prostate cancer.

## 1. Introduction

Prostate cancer (PC) remains a major global health concern and is the second most frequently diagnosed cancer in men and the fifth leading cause of cancer-related mortality worldwide [1]. The development and progression of PC depend on androgen signaling, which is a key therapeutic target in the early stages of the disease [2]. However, new global population and epidemiological dynamics appear to increase the prevalence of advanced forms of PC, such as metastatic prostate cancer (mPC) and metastatic castration-resistant prostate cancer (mCRPC), which are prone to therapeutic failure [3,4,5,6].

For patients with mPC and mCRPC, standard chemotherapy relies on taxane-based agents such as docetaxel (DCT) to improve the effectiveness of systemic treatment [7]. Despite its clinical benefits, DCT therapy yields only modest improvements in patient survival and is frequently associated with systemic toxicity in non-target tissues [8]. Moreover, many patients develop chemoresistance, which remains a major obstacle to the long-term efficacy of taxane-based therapies [9].

Highly metastatic and chemoresistant cancer cells are particularly well adapted to cope with intrinsic or therapy-induced oxidative stress. These cells often increase their antioxidant capacity, reprogram their metabolic networks, and shift toward glycolytic metabolism under hypoxic conditions, while suppressing mitochondrial respiration even in the presence of oxygen [10,11]. Given these observations, biologically active molecules that modulate redox signaling and metabolic pathways represent promising therapeutic alternatives for treating prostate cancer. Such strategies may simultaneously enhance tumor sensitivity to chemotherapy while limiting damage to non-malignant tissues [12].

Dimethyl fumarate (DMF) is an FDA-approved drug used to treat multiple sclerosis and other inflammatory disorders. As a derivative of the Krebs cycle intermediate fumarate, DMF is an electrophilic molecule that covalently modifies reactive cysteine residues on key proteins via succination [13]. In non-cancer models, DMF has demonstrated potent antioxidant and cytoprotective properties, largely attributed to activation of the nuclear factor erythroid 2-related factor 2 (Nrf2)-dependent signaling pathway and the subsequent upregulation of downstream antioxidant enzymes such as heme oxygenase-1 (HO-1) and NAD(P)H: quinone oxidoreductase 1 (NQO1) [14,15]. Beyond the Nrf2 axis, DMF directly modulates the glutathione (GSH) antioxidant system: it conjugates with GSH, causing acute intracellular GSH depletion, while GSH levels may subsequently recover and rise above baseline through Nrf2-mediated transcriptional upregulation of glutathione biosynthetic enzymes [16]. Furthermore, DMF has been reported to influence mitochondrial physiology, including mitochondrial biogenesis programs involving PGC-1α, NRF1, and TFAM, as demonstrated both in vitro and in vivo in mice and humans [17].

Importantly, accumulating evidence indicates that DMF also exerts anticancer effects through specific metabolic and redox mechanisms that extend well beyond Nrf2 modulation. At the metabolic level, DMF succinates and inactivates the catalytic cysteine of glyceraldehyde 3-phosphate dehydrogenase (GAPDH), thereby suppressing aerobic glycolysis [13]. Structural studies show that this covalent modification blocks NAD co-substrate binding via steric hindrance [18]. DMF has also been shown to promote the oxidative pentose phosphate pathway via glucose-6-phosphate dehydrogenase (G6PD), altering NADPH availability and redirecting carbon flux away from glycolysis [19]. In cancer cells, DMF can simultaneously depress mitochondrial respiration and aerobic glycolysis, inducing a metabolic crisis [20]. Additionally, metabolomic profiling of DMF-treated patients has revealed increased levels of tricarboxylic acid (TCA) cycle intermediates—including fumarate and succinate—and their associated metabolites (succinyl-carnitine and methyl succinyl-carnitine), suggesting a reversal of flux through the succinate dehydrogenase (SDH) complex [21].

At the redox level, high concentrations of DMF deplete intracellular GSH in cancer cells, leading to increased accumulation of reactive oxygen species (ROS), loss of mitochondrial membrane potential, and activation of stress-responsive MAPK cascades (JNK, p38, ERK), ultimately triggering cell death [22]. Notably, these pro-oxidant and cytotoxic effects in malignant cells contrast with the cytoprotective response observed in non-tumorigenic cells, where the same concentrations of DMF activate Nrf2, reduce ROS, and increase GSH levels [23].

Previous studies have indicated that the anticancer activity of DMF is highly dose-dependent. Saidu et al. demonstrated that high concentrations of DMF induce cytotoxicity in several tumor cell lines, in part by reducing Nrf2 nuclear translocation and decreasing DJ-1 expression, a key stabilizer of the NRF2 protein. Conversely, low concentrations of DMF may promote Nrf2 activation and stimulate antioxidant and detoxification responses [14]. These observations highlight the context-dependent biological activity of DMF and suggest that modulating the Nrf2–redox axis may be a therapeutically exploitable mechanism in cancer.

In prostate cancer, DMF has recently emerged as a promising candidate for drug repurposing strategies. Experimental evidence suggests that DMF may exert antitumor activity while simultaneously providing protective effects against chemotherapy-associated oxidative stress, including docetaxel-induced toxicity [24]. However, despite these promising observations, the molecular basis and therapeutic implications of DMF–DCT interactions remain largely unexplored, particularly in advanced prostate cancer [25]. Based on these observations, we hypothesized that DMF could serve as a context-dependent modulator of the DCT response in prostate cancer, acting on both metabolism and redox status. Therefore, the present study aimed to: (i) assess the cytotoxic profile of low-dose DMF and DCT in non-malignant prostate epithelial cells; and (ii) investigate whether DMF–DCT combinations elicit phenotype-dependent metabolic, redox, and cell death responses in prostate cancer cell models with varying androgen sensitivity.

## 2. Results

### 2.1. Low-Dose Dimethyl Fumarate Reduces Prostate Cancer Cell Biomass While Preserving Metabolic Activity and Maintaining Low Toxicity in Normal Prostate Cells

Initial experiments were conducted to determine the individual half-maximal inhibitory concentrations (IC50) of DMF and DCT in RWPE-1, PC-3, and LNCaP cell lines. Crystal violet (CV) staining was used to estimate adherent cell biomass/cell density in cultures harvested at 24, 48, and 72 h after treatment. Because CV absorbance was normalized to untreated cultures, considered as 100%, this assay was used as an indirect readout of treatment effects on cell viability and survival. Dose–response curves for each condition are presented in Figure S1, and IC50 values were calculated by nonlinear regression analysis. The resulting IC50 values for each compound, cell line, and exposure time are summarized in Table S1.

**Figure 1:**
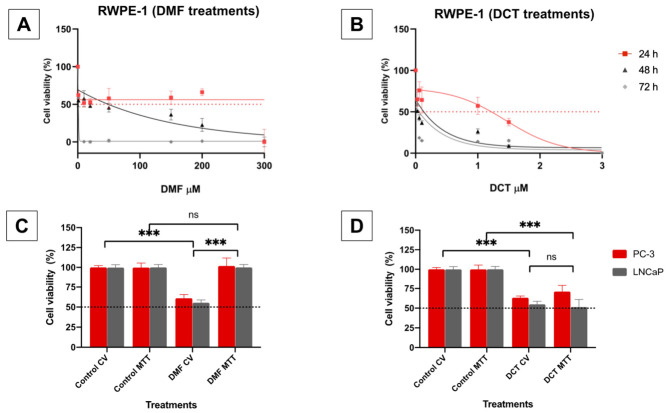
DMF reduces prostate cancer cell viability while preserving cellular metabolic activity, whereas DCT reduces both parameters. Cell density in RWPE-1 cells was evaluated using the crystal violet (CV) assay after treatment with increasing concentrations of (**A**) dimethyl fumarate (DMF, 0–300 µM) and (**B**) docetaxel (DCT, 0–3 µM) for 24, 48, and 72 h to generate dose–response curves and estimate IC50 values. Dose–response curves represent the mean ± SEM from at least three independent experiments (*n* = 3). To investigate the relationship between cell viability and cellular metabolic activity in prostate cancer cells, PC-3 and LNCaP cells were treated for 48 h with their respective IC50 concentrations of DMF or DCT. Cellular metabolic activity was evaluated using the MTT reduction assay. For CV and MTT measurements, bars represent values normalized to the corresponding control group and are shown as mean ± SEM from three independent experiments. (**C**) Comparison of cell viability and cellular metabolic activity in PC-3 and LNCaP cells treated for 48 h with their respective DMF IC50 concentrations (PC-3: 47.5 µM; LNCaP: 109.8 µM). (**D**) Comparison of cell viability and cellular metabolic activity in PC-3 and LNCaP cells treated for 48 h with their respective DCT IC50 concentrations (PC-3: 0.010 µM; LNCaP: 0.132 µM). Statistical significance was determined using Dunnett’s post hoc test comparing treated groups with their respective controls. *** *p* < 0.001; ns, not significant.

We first evaluated whether RWPE-1 cells, used as a model of non-malignant prostate epithelium, could tolerate the proposed dose-rationale regimen. The results for RWPE-1 are summarized in Figure 1A,B. After both 24- and 48-h treatments, CV-derived adherent cell viability remained above 50% across the tested concentration ranges of DMF and DCT. These findings indicate that the selected dose regimen exerts limited cytotoxic effects on non-malignant prostate epithelial cells and supports the relatively low toxicity profile of the DMF/DCT exposure model.

Having established that the selected dose regimen exhibited limited toxicity toward non-malignant prostate epithelial cells, we subsequently evaluated its effects in prostate cancer cells. PC-3 and LNCaP cells were exposed for 48 h to their respective IC50 concentrations of DMF or DCT. CV staining was used to estimate adherent cell biomass/cell density, whereas the MTT assay was used to assess cellular metabolic reducing activity. Because MTT reduction depends on the capacity of viable cells to reduce tetrazolium salts, it was interpreted as an indirect indicator of cellular metabolic activity rather than as a direct measure of cell number.

Individual treatments with DMF and DCT significantly reduced cell viability in PC-3 and LNCaP cells, as determined by CV staining (Figure 1C,D, *p* < 0.001). Notably, despite the marked reduction in cell biomass induced by DMF, MTT reduction remained comparable to that observed in the corresponding control groups (Figure 1C). This finding indicates that the metabolic activity of the remaining viable cell population was largely preserved under DMF exposure, consistent with previous in vitro observations reported for other Nrf2 activators, including sulforaphane [26].

In contrast, DCT treatment significantly reduced both cell density and MTT reduction (Figure 1D, *p* < 0.001), indicating a concurrent decline in viable cell number and overall cellular metabolic activity.

Taken together, these findings indicate that DMF reduces prostate cancer cell viability, as reflected by decreased CV-derived adherent cell biomass, while preserving MTT-derived metabolic reducing activity. The divergence between CV- and MTT-derived responses suggests that DMF affects adherent cell density and/or survival differently from DCT, resulting in a partial dissociation between cell biomass and cellular metabolic activity under the experimental conditions evaluated.

### 2.2. DMF–DCT Combination Displays Phenotype and Dose-Dependent Pharmacological Interactions with an Overall Decreased Cell Viability in Advanced Prostate Cancer

**Figure 2:**
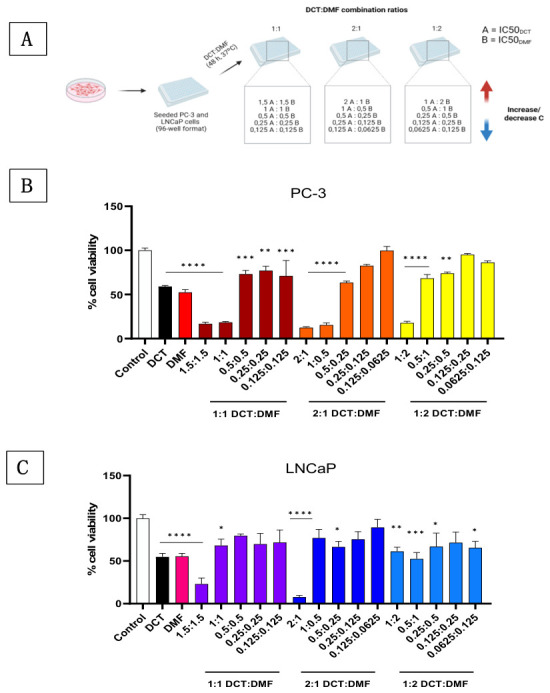
Dose–ratio-dependent effects of DMF–DCT combinations on prostate cancer cell viability. (**A**) Experimental design of combination treatments. PC-3 and LNCaP cells were seeded in 96-well plates and treated for 48 h with dimethyl fumarate (DMF) and docetaxel (DCT) using fixed-ratio combinations (1:1, 2:1, and 1:2) based on their respective IC50 values (A = IC50DCT; B = IC50DMF). Serial dilutions were used to generate concentrations above and below the IC50 for each ratio. (**B**) Effects of DMF–DCT combinations on PC-3 cell viability. Cells were treated for 48 h, and viability was assessed by CV staining. Data are expressed as the percentage of cell viability relative to the untreated control. Combination treatments show a dose- and ratio-dependent response, with greater cytotoxic effects at higher concentrations, particularly at the 1:1 ratio. (**C**) Effects of DMF–DCT combinations on LNCaP cell viability. Under the same experimental conditions, LNCaP cells exhibited a heterogeneous response to combination treatments, with marked reductions in viability at higher concentrations and variable effects at intermediate and lower doses, depending on the drug ratio. Data are presented as mean ± SEM from at least three independent experiments (*n* = 3). Statistical significance was determined relative to control (* *p* ≤ 0.05, ** *p* ≤ 0.01, *** *p* ≤ 0.001, **** *p* ≤ 0.0001).

To evaluate the pharmacological interaction between DMF and DCT, we used the experimental design summarized in Figure 2A, based on previously calculated IC50 increase/decrease ratios for each compound (DCT and DMF). The results of the cell viability calculations by CV for PC-3 and LNCaP are summarized in Figure 2B,C, respectively. This strategy enabled assessment of dose-dependent combinatorial effects across a wide pharmacological window in both PC-3 and LNCaP cells.

In PC-3 cells (Figure 2B), single-agent treatments showed the expected reduction in viability, but DMF alone exhibited a pronounced cytotoxic effect. Combination treatments revealed heterogeneous responses, depending on both the ratio and the concentration. At the 1:1 ratio, higher concentrations (1.5:1.5 and 1:1) resulted in a marked decrease in viability, reaching values below 25%, indicating strong combined cytotoxicity. However, as concentration decreased, viability progressively increased, yet significant cell-viability loss persisted, maintaining combinatorial efficacy at lower doses. A similar trend was observed in the 2:1 ratio (high DCT vs. DMF). However, the overall effect persisted until the combinations reached 0.5:0.25 (an intermediate combination ratio), with partial reductions in viability at lower doses. Similarly, the 1:2 ratio (high DMF vs. DCT) showed a significant cytotoxic profile overall, with intermediate combination ratios maintaining viability above ~50–60%, suggesting that increasing DMF’s relative contribution might sustain proportionally enhanced cytotoxicity in this cell line.

In LNCaP cells (Figure 2C), a distinct response pattern was observed. While single-agent treatments again reduced viability, combination treatments showed greater variability in their effects. At the 1:1 ratio, the highest concentration (1.5:1.5) induced a strong reduction in viability (~20–30%). However, at 1:1, intermediate, and lower concentrations, partial recovery of viability was observed, suggesting a non-linear dose–response relationship. Notably, at the 2:1 ratio, a biphasic response was evident, with certain intermediate concentrations (e.g., 0.5:0.25 and 0.25:0.125) exhibiting reduced cell density compared with lower doses, suggesting possible adaptive responses. Interestingly, the 1:2 ratio maintained a significant reduction in cell viability across most concentrations, with viability generally remaining above 50%, suggesting the context-dependent contribution of DMF-dominant conditions to metabolic effects in LNCaP cells.

The combination index (CI) analysis summarized in Table S2 showed a prevalence of antagonistic responses, with distinct regions of synergy that varied by dose and ratio, consistent with the viability profiles in Figure 2. The strongest synergistic interactions were typically confined to higher concentration ranges and specific ratio windows in both PC-3 and LNCaP cells, especially around equimolar or attenuated DCT conditions, which correlated with the most pronounced decreases in cell viability. DMF-dominant ratios also followed a similar pattern. Notably, the ratio-dependent CI distribution indicates complex pharmacodynamic interactions, suggesting that the cytotoxic efficacy of the DMF–DCT combination might depend on dose optimization.

We also examined individual dose–response curves alongside isobolographic analysis to confirm the non-linear distribution of DMF and DCT combinations. Results in PC-3 cells revealed non-constant relative potency for DMF and DCT treatments (Figure S2A), with significant differences in cell viability (*p* = 0.0019), yielding a curve-fitted additive isobole (Figure S2C). In contrast, LNCaP cells showed constant relative potency (Figure S2B) due to similar Hill slopes, producing a linear isobole (Figure S2D). All data for isobole construction were obtained according to the method proposed by Tallarida [27,28], which confirmed the cell context-dependent interaction of DMF-DCT, favoring greater DMF-induced DCT sensitization in PC-3 than in LNCaP cells. In fact, major DCT:DMF ratios, such as 1:1, 2:1, and 1:2, showed synergistic effects on cell viability in PC-3-treated cells.

Therefore, DMF should not be interpreted as a uniform sensitizer across both prostate cancer models; rather, pharmacological sensitization was most evident in PC-3 cells, which required higher concentration ratios to ensure synergism. However, a significant dose-sparing effect was found for both agents, especially for DCT across all tested ratios, according to dose reduction index (DRI) analysis (Table 1). The 2:1 DCT: DMF ratio yielded the largest DCT dose reduction (DRI ≈ 52.67) in PC-3 cells, suggesting that comparable cytotoxic effects can be achieved with significantly lower docetaxel concentrations when combined with DMF. Similarly, the 1:1 ratio showed a strong reduction capacity (DRI ≈ 42.44), while the 1:2 ratio displayed a comparatively lower but still relevant effect (DRI ≈ 22.03). In contrast, DMF showed more modest DRI values across conditions, with the highest reduction observed at the 1:2 ratio (DRI ≈ 14.20). Our results suggest that DMF–DCT combinations preferentially potentiate docetaxel activity, supporting DCT-dominant regimens as a more successful approach to dose optimization, based on IC50 values and higher concentration ranges.

### 2.3. DMF-DCT Combination Promotes Differential Metabolic Alterations Accompanied by Glutathione-Associated Changes in Advanced Prostate Cancer

For the following experiments, DMF-DCT combinations were prepared at a 1:1 ratio and at half the IC50-derived concentrations to reflect the differential sensitivity of PC-3 cells. This ensured sufficient cell biomass for experiments aimed at elucidating molecular mechanisms.

To corroborate the significance of the sustained mitochondrial activity decoupled from cell viability reduction, we performed complementary assays to determine whether prostate cancer cells exposed to the DMF-DCT combination adopt a distinct oxidative metabolic phenotype, potentially reflecting a redistribution of carbon flux toward mitochondrial metabolism. Notably, increased mitochondrial activity coupled with sustained intracellular ROS levels has been associated with enhanced therapeutic vulnerability in prostate cancer models [29]. In line with this, our previous findings demonstrated that metabolic and glutathione-associated changes without effective antioxidant rescue sensitizes prostate cancer cells with differential metastatic potential [30].

**Figure 3:**
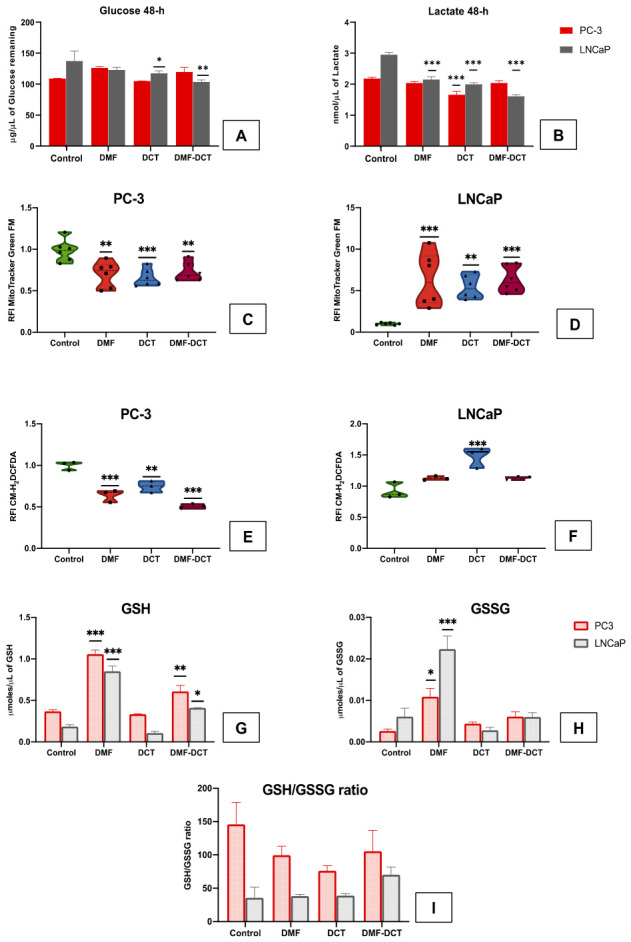
The DMF-DCT combination modified glycolytic activity, lactate production, mitochondrial mass and oxidative stress in prostate cancer cells. Glucose consumption (**A**) and lactate production (**B**) measured at 48 h in LNCaP and PC-3 cell lines. Mitochondrial mass in PC-3 cells (**C**), Mitochondrial mass in LNCaP cells (**D**) ROS production induced by treatments in PC-3 (**E**) ROS production induced by treatments in LNCaP (**F**), GSH levels (**G**), GSSG levels (**H**), and GSH/GSSG ratio (**I**) measured at 48 h in LNCaP, and PC-3 cell lines. Observed data corresponds to the normalization of raw values compared to the control value (untreated cells) in each experiment. Statistical significance was determined by comparison with the respective control (* *p* ≤ 0.05; ** *p* ≤ 0.01; *** *p* ≤ 0.001). Data are represented as mean ± standard error of the mean (SEM) from three independent experiments. GSH and GSSG levels were determined in standardized extracts prepared from 1 × 10^7^ cells per condition using identical extraction and dilution volumes. The GSH/GSSG ratio was calculated from the corresponding GSH and GSSG values for each condition.

Upon treatment, a marked metabolic shift was observed. After 48 h of exposure, only LNCaP cells displayed a significant reduction in residual glucose levels, indicating enhanced glucose uptake under DMF-DCT treatment (*p* ≤ 0.01) (Figure 3A). Notably, all treatment conditions significantly reduced lactate production in LNCaP cells relative to untreated controls, with the DMF-DCT combination eliciting the most pronounced decrease (*p* ≤ 0.001) (Figure 3B).

To further validate the mitochondrial oxidation differential profiles induced by DMF-DCT treatments, we quantified mitochondrial mass across cell lines. Results from treated cells and their mitochondrial masses are summarized in Figure 3C,D. In PC-3 cells, all treatments significantly reduced mitochondrial mass, particularly DMF–DCT (*p* < 0.01), suggesting mitochondrial selective depuration or loss. In contrast, LNCaP cells exhibited a significant increase in mitochondrial mass, especially under DMF–DCT treatment (*p* ≤ 0.001), consistent with compensatory remodeling in response to metabolic stress.

Given these divergent mitochondrial adaptations, we next evaluated whether ROS-driven responses were associated with treatment-induced metabolic reprogramming. Upon treatment, PC-3 cells showed a consistent reduction in ROS levels across all conditions (Figure 3E), further supporting their limited metabolic responsiveness. In contrast, LNCaP cells exhibited increased ROS levels, with DCT treatment inducing a significant elevation (*p* ≤ 0.001), while DMF–DCT maintained ROS at sustained levels without significant reduction (*p* = 0.25) (Figure 3F). This sustained ROS phenotype, together with increased mitochondrial mass, suggests a state of mitochondrial-driven oxidative stress rather than efficient redox adaptation.

Despite ROS accumulation, antioxidant responses might be insufficient to counterbalance the pro-oxidant effects induced by DMF–DCT, particularly in LNCaP cells. To address this, we quantified GSH and GSSG levels following treatment. DMF alone significantly increased GSH levels in both PC-3 and LNCaP cells (*p* ≤ 0.001) (Figure 3G). Similarly, DMF–DCT treatment elevated GSH content, with a more pronounced effect in PC-3 cells, followed by LNCaP cells (Figure 3G). GSSG levels were also modulated, with DMF treatment significantly increasing GSSG in LNCaP cells (*p* ≤ 0.001) and, to a lesser extent, in PC-3 cells (*p* ≤ 0.05) (Figure 3H). However, analysis of the GSH/GSSG ratio showed no statistically significant differences across experimental conditions in either cell line (Figure 3I). In PC-3 cells, DMF and DCT showed a tendency to reduce the GSH/GSSG ratio compared with untreated controls, whereas the DMF-DCT combination partially restored this ratio without reaching control levels. In LNCaP cells, the DMF-DCT combination tended to increase the GSH/GSSG ratio compared with the individual treatments, although this change was not statistically significant. Therefore, the glutathione data indicate treatment-induced remodeling of the glutathione pool, but do not support a definitive reductive stress phenotype.

### 2.4. DMF–DCT Induces Divergent Caspase-Linked Apoptotic Responses in Advanced Metastatic Prostate Cancer Cells

**Figure 4:**
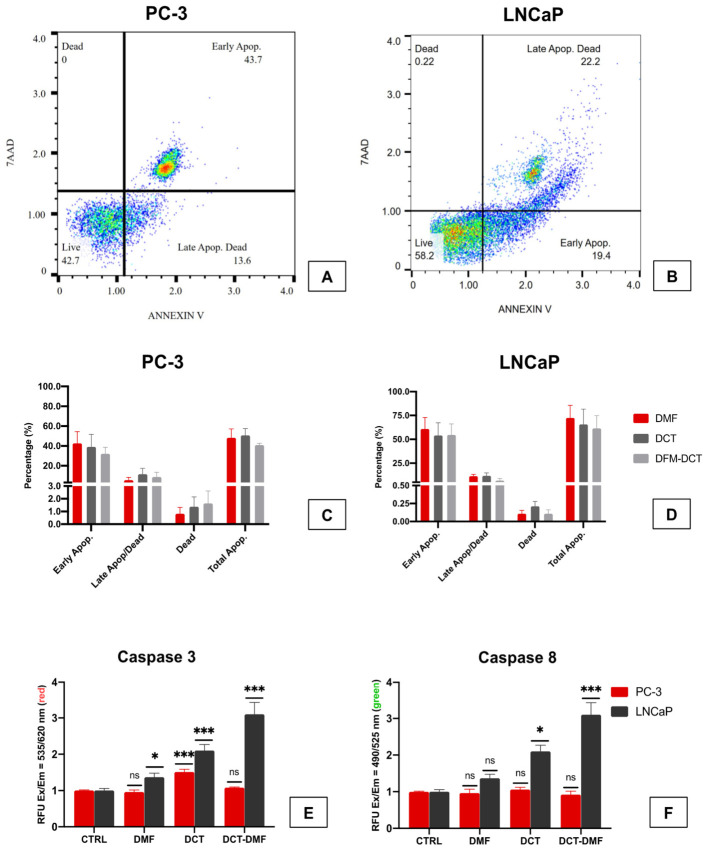
DMF–DCT induces divergent apoptotic responses in prostate cancer cells. Representative Annexin V scatterplots are shown for PC-3 (**A**) and LNCaP (**B**) cells treated with the DMF–DCT combination for 48 h. Quantification of apoptotic events determined by Annexin V staining is shown for PC-3 (**C**) and LNCaP (**D**) cells. Combined DMF–DCT treatment promoted caspase-3 (**E**) and caspase-8 (**F**) activation in LNCaP cells, but not in PC-3 cells. No caspase-9 activation was detected under any treatment conditions in either prostate cancer cell line (Figure S3). Results are expressed as the mean ± SEM from at least three independent experiments. Data corresponds to normalized values relative to the respective untreated control group. Statistical significance was determined by comparison with the respective control (* *p* ≤ 0.05; *** *p* ≤ 0.001).

Annexin V assay data for PC-3 cells are summarized in Figure 4A,C. Overall, total apoptotic events in PC-3 cells remained below 50% across all treatments. DMF and DCT showed comparable effects, while the DMF–DCT combination induced approximately 40% total apoptotic events. PC-3-treated cells also showed a predominance of early apoptotic events, although they did not exceed 40% of the total population. Late apoptotic events consistently accounted for less than 10% of total cell events, suggesting a limited progression toward advanced stages of apoptosis (Figure 4C).

In contrast, LNCaP cells exhibited a markedly stronger apoptotic response. Annexin V assay data for LNCaP cells are shown in Figure 4B,D. DMF treatment alone induced approximately 70% total apoptotic events, whereas DCT and the DMF–DCT combination accounted for around 65%. Early apoptotic events predominated, representing about 55% of total events in DMF-treated cells and approximately 50% in both DCT and DMF–DCT conditions. Late apoptotic events were reduced to ~20% or less across all treatments. These results confirm a sustained and pronounced apoptotic response in LNCaP cells.

To further characterize the apoptotic mechanisms involved, we evaluated caspase activation. In LNCaP cells, caspase-3 and caspase-8 activation increased significantly after DMF–DCT treatment (*p* < 0.001), with a stronger effect compared to individual treatment, suggesting activation of apoptosis-related signaling (Figure 4E,F). However, no caspase-9 activation was detected under any treatment condition (Figure S3), indicating that canonical caspase-9-dependent intrinsic apoptosis was not confirmed.

In contrast, PC-3 cells showed minimal activation of apoptotic signaling pathways (Figure 4E,F). Caspase-3 activation was only detected following DCT treatment (*p* < 0.001), whereas DMF and DMF–DCT did not induce significant changes. Similarly, caspase-8 levels remained unaltered across all treatments. As observed in LNCaP cells, no activation of caspase-9 was detected in PC-3 cells (Figure S3). Together, these data suggest that the DMF–DCT combination induces a stronger caspase-linked apoptotic phenotype in LNCaP cells, whereas PC-3 cells exhibit reduced viability with limited apoptotic engagement.

### 2.5. DMF-DCT Combination Modulated Differential SOD2 and LDHA Expression, Displaying Mitochondrial Dysfunction in Metastatic Prostate Cancer

To determine the modulatory effect of DMF, DCT, and DMF-DCT on the nuclear translocation of Nrf2 and HIF1-*α* transcription factors, we measure *SOD2* and *LDHA* expression using RT-qPCR. We wanted to assess whether glycolytic and redox modulation in PC-3 and LNCaP cells can also be reversed at the transcriptomic level. *SOD2* is part of the antioxidant gene response triggered by Nrf2, while *LDHA* is part of the hypoxia-response element activated by HIF1-*α* in tumor cells. The different treatments modulated the relative expression of *LDHA* and *SOD2*.

**Figure 5:**
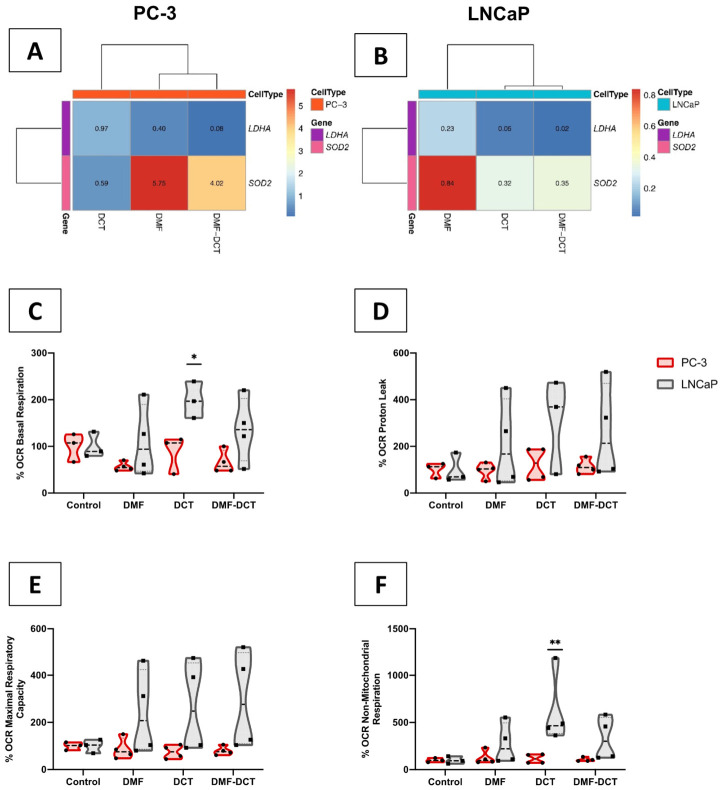
The DMF-DCT combination induces differential *SOD2* and *LDHA* expression with mitochondrial dysfunctions depending on the differential androgen response in advanced prostate cancer. Relative expression heatmaps of *LDHA* and *SOD2* in PC-3 (**A**) and LNCaP (**B**) cells after 48 h of treatment. Heatmaps were generated from normalized RT-qPCR relative quantification (RQ) values. Sample-to-sample clustering was performed using Euclidean distance calculated with the dist function in R (R stats package version 4.5.2), and treatment relationships are shown as dendrograms. Oxygen consumption parameters were measured after 48 h of treatment and are shown as percentage OCR for basal respiration (**C**), proton leak (**D**), maximal respiration (**E**), and non-mitochondrial respiration (**F**) in LNCaP and PC-3 cells. Results are expressed as the mean ± SEM from three independent biological experiments (*n* = 3). Statistical significance was determined by comparison with the respective control; * *p* ≤ 0.05 and ** *p* ≤ 0.01.

In PC-3 cells, DMF and DMF-DCT induced a similar gene expression pattern, with both maintaining *SOD2* overexpression. In contrast, *LDHA* showed a reduced expression pattern, evidenced by an *LDHA/SOD2* cluster grouped by Euclidean distances (Figure 5A), suggesting a shift toward a redox adapted but less glycolytic phenotype.

In contrast, in LNCaP cells, the *SOD2* overexpression induced by DMF was attenuated in both DCT and DMF–DCT groups, accompanied by a concomitant downregulation of *LDHA* (Figure 5B). Together with the increased ROS levels, higher mitochondrial mass, and altered respiratory parameters observed in this cell line, these data suggest that LNCaP cells undergo a mitochondrial stress-associated response in which the antioxidant-related transcriptional response may be insufficient to counterbalance treatment-associated oxidative stress.

Finally, to assess whether changes in mitochondrial mass and oxidative stress can be related with mitochondrial dysfunctions that explain LNCaP differential sensitive effects, respirometry data for basal and treated cells were obtained, using measured oxygen consumption rate. Furthermore, LNCaP was the only cell line responsive to DCT, which induced a significant increase in basal mitochondrial respiration (Figure 5C; * *p* ≤ 0.05), proton leak (Figure 5D), maximal respiratory capacity (Figure 5E), and non-mitochondrial respiration (Figure 5F; ** *p* ≤ 0.01). Although not reaching statistical significance, DMF–DCT-treated cells showed a consistent upward trend across these parameters, suggesting that the DCT-driven effect is partially maintained within the combination. In contrast, no differential oxygen consumption was observed in PC-3 cells treated with either DMF or DMF–DCT.

Our findings support the presence of enhanced mitochondrial oxidation occurring under conditions of bioenergetic stress rather than improved mitochondrial efficiency, with LNCaP cells displaying a heightened sensitivity to this metabolic perturbation.

## 3. Discussion

The effects of DMF have been linked to modulation of canonical signaling pathways and to its ability to covalently bind key proteins involved in transcriptional regulation and redox balance, as well as other relevant cellular regulators. These properties confer upon this molecule a leading role as a potential therapeutic alternative in various types of cancer [25]. However, studies specifically exploring its effects on prostate cancer are still limited Although DMF has demonstrated promising biological activity in this context, its development as a therapeutic strategy has not been extensively explored in subsequent studies [31,32].

Within this framework, the present work from our group and previous work by collaborators both goes beyond helping to reignite the discussion regarding the use of DMF as a chemotherapeutic alternative in prostate cancer. Our findings indicate that, while IC50-based low-concentration DMF-DCT regimens and those dominated by DMF exhibit conserved efficacy and tend toward antagonistic interactions, the most favorable combination windows are observed under conditions of higher exposure and in regimens balanced or enriched with docetaxel, which are mostly synergistic, consistent with previous research [24]. Our work shows promising results, as PC-3 cells exhibit differential sensitivity to the DMF-DCT combination, and their synergistic interactions can be strategically exploited to achieve substantial dose reductions, particularly for docetaxel. The latter might be clinically relevant, given that DCT-based chemotherapy is frequently limited by dose-dependent toxicity and the emergence of resistance.

In addition, we have observed differential effects of DMF and DCT in our experimental paradigm, with both treatments showing low toxicity in normal prostate epithelial cells. This finding is particularly relevant, as it suggests a potential margin for therapeutic selectivity for the DMF–DCT combination. Like our results DMF concentrations below 25 μM increase cytoprotective effects in ovarian carcinoma cells by modulating the Nrf2 antioxidant pathway [14]. Our results are encouraging and support the need to explore further cytoprotection models and more complex systems to validate and extend these observations to preclinical settings.

However, the translational interpretation of DMF effects requires caution. The present study was designed as an in vitro mechanistic approach, and the DMF concentrations used to define cytotoxic and combination-response windows should not be interpreted as directly equivalent to clinically achievable systemic exposure. In vivo, DMF is rapidly hydrolyzed to its active metabolite monomethyl fumarate (MMF), whose reported plasma exposure is generally lower than several of the DMF concentrations evaluated in our dose–response experiments [33,34]. In contrast, the DCT concentrations used in this study are more closely aligned with pharmacodynamic ranges previously explored for taxane-based responses, which supports the relevance of evaluating whether fumarate-mediated metabolic modulation can influence DCT sensitivity [35]. Accordingly, the main value of our combination data lies not in reproducing plasma-equivalent DMF/MMF exposure, but in identifying phenotype-dependent metabolic–redox vulnerabilities that may be exploited to modulate DCT responses. Future studies should directly compare DMF and MMF at clinically relevant concentrations. Our findings should be considered hypothesis-generating and mechanism-oriented, providing a rationale for future preclinical studies rather than direct evidence of immediate clinical translatability.

**Figure 6:**
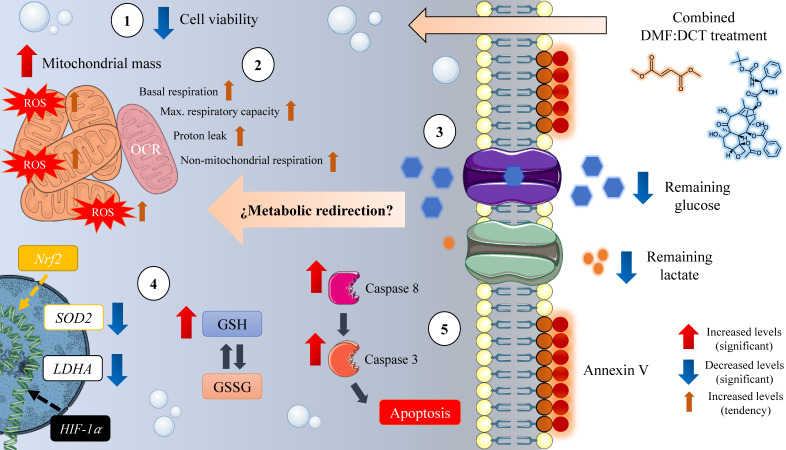
DMF–DCT induces metabolic alterations accompanied by glutathione-associated changes and a mitochondrial stress-associated, caspase-linked apoptotic phenotype in LNCaP cells. (**1**) Combined DMF–DCT treatment significantly reduces cell viability in LNCaP cells, concomitant with a compensatory increase in mitochondrial metabolic activity. (**2**) This response is characterized by increased mitochondrial mass and greater mitochondrial respiratory engagement, as reflected by elevated basal and maximal respiratory capacity. However, the concomitant increase in proton leak indicates that mitochondrial respiration occurs under conditions of bioenergetic stress. In parallel, DMF-DCT sustains elevated ROS levels, maintaining LNCaP cells under persistent oxidative stress. (**3**) Consistent with this metabolic reprogramming, increased extracellular glucose consumption and reduced lactate levels suggest a redirection of pyruvate flux away from lactate production and toward mitochondrial oxidation. (**4**) This sustained mitochondrial reliance appears to be accompanied by a transcriptionally insufficient antioxidant response, characterized by reduced *SOD2* and *LDHA* expression. Despite a compensatory increase in GSH levels, these responses are inadequate to suppress ROS accumulation, while concurrently reflecting a diminished glycolytic phenotype consistent with a partial reversal of the Warburg effect. (**5**) Caspase-8 and caspase-3 activation, together with Annexin V positivity, supports the induction of a caspase-linked apoptotic response associated with mitochondrial metabolic stress. However, because caspase-9 activation was not detected, canonical intrinsic mitochondrial apoptosis was not confirmed. Overall, this model suggests that DMF–DCT-induced metabolic alterations accompanied by glutathione-associated changes promoting a pro-oxidant, bioenergetically stressed state that enhances therapeutic vulnerability in LNCaP cells. The large upper arrow indicates the cellular entry of DMF and DCT, whereas the central pale arrow represents the proposed metabolic redirection induced by the combined treatment. Dashed arrows indicate transcriptional regulation mechanisms yet to be elucidated.

**Figure 7:**
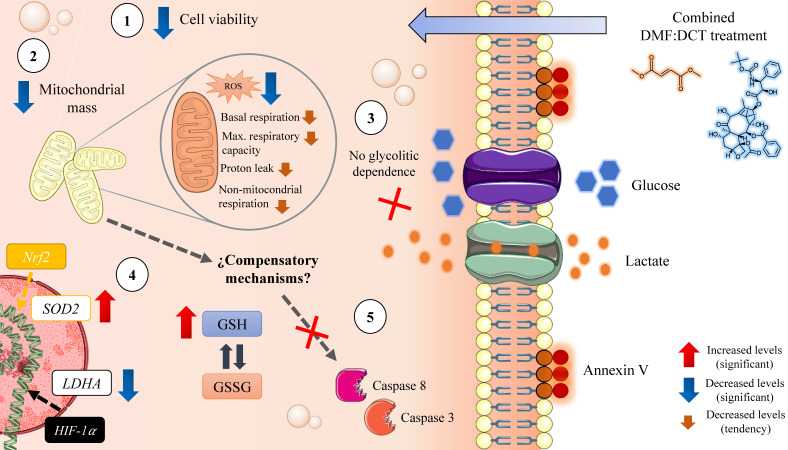
DMF-DCT induces a low-glycolytic profile with concomitant cytotoxicity and glutathione-associated changes, along with mitochondrial clearance in PC-3 cells. (**1**) Combined DMF-DCT treatment reduces cell viability in PC-3 cells, consistent with a cytotoxic response. (**2**) This effect is accompanied by decreased mitochondrial mass, reduced ROS levels, and a global reduction in respiratory activity, including basal respiration, maximal respiratory capacity, proton leak, and non-mitochondrial respiration. Together, these results might indicate mitochondrial clearance. (**3**) Despite decreased *LDHA* expression, lactate production remains sustained, while extracellular glucose levels increase, indicating a lack of effective metabolic redirection and suggesting that PC-3 cells maintain metabolic flexibility without strict dependence on glycolysis. (**4**) Antioxidant defenses might be preserved due to increased *SOD2* expression and elevated GSH levels, despite cell viability decreases. (**5**) Annexin V detection indicates only a moderate induction of apoptosis, consistent with limited engagement of cell death pathways. Collectively, these findings suggest that DMF-DCT promotes moderate metabolic reprogramming, reducing glycolytic dependence and increasing mitochondrial dysregulation in PC-3 cells, which may underlie compensatory mechanisms despite DMF-DCT administration. The large blue arrow indicates the cellular entry of DMF and DCT. Red crosses indicate pathways or responses that appear to be blocked or not activated, including glycolytic dependence and downstream apoptotic signaling. Dashed arrows indicate transcriptional regulation mechanisms yet to be elucidated.

Rather than acting as a conventional cytotoxic co-drug or a uniform docetaxel chemosensitizer, our findings support DMF as a context-dependent modulator of docetaxel response in prostate cancer cells. The most relevant finding is that, depending on the prostate cancer phenotype, low-dose DMF-DCT decreases viability through noticeably different biological trajectories. In LNCaP cells, our results indicate that the DMF-DCT combination reduces cell viability via metabolic alterations accompanied by glutathione-associated changes and mitochondrial stress-induced apoptosis (Figure 6). On the other hand, PC-3 showed differential sensitivity to DMF-DCT coadministration, accompanied by differential moderated metabolic reprogramming, with mitochondrial clearance and limited glycolytic dependence. However, PC-3’s inherent cell resistance limited the apoptotic response (Figure 7).

The differential action of the DMF-DCT combination in LNCaP and PC-3 cells may also be interpreted considering glutathione remodeling. DMF alone increased both reduced and oxidized glutathione pools in both prostate cancer cell lines, suggesting activation of the glutathione system rather than a simple antioxidant or pro-oxidant effect. This response is consistent with the electrophilic nature of DMF, which can modify redox programs in cell including NADPH metabolism, and glutathione homeostasis [36]. Interestingly, when DMF was combined with DCT, GSH remained elevated whereas GSSG approached control levels in both prostate cancer cell lines, suggesting a shift from overt glutathione oxidation toward glutathione-associated buffering or recycling. This distinction is biologically relevant, since enhanced GSH availability may either represent an adaptive antioxidant response to treatment-induced stress or attenuate ROS-dependent cytotoxicity and influence taxane efficacy in a phenotype-dependent manner [37,38].

Although no statistically significant differences were observed in the GSH/GSSG ratio, the combined analysis of reduced and oxidized glutathione levels, together with ROS, mitochondrial mass, and metabolic assays, provides relevant insights into the phenotype-dependent adaptations induced by DMF-DCT. In PC-3 cells, the glutathione-associated response occurred together with reduced ROS levels, lower mitochondrial mass, and a non-significant decrease in the GSH/GSSG ratio, supporting an adaptive glutathione modulation coupled to mitochondrial contraction rather than definitive reductive stress or complete bioenergetic preservation [39,40,41]. Conversely, in LNCaP cells, increased GSH availability occurred despite sustained ROS levels, increased mitochondrial mass, altered respiratory parameters, and stronger apoptotic activation, suggesting that antioxidant compensation was insufficient to prevent mitochondrial stress-associated cell death. Therefore, DMF-DCT appears to remodel glutathione homeostasis in a phenotype-dependent manner, reinforcing the notion that this combination acts as a metabolic modulator rather than as a uniform docetaxel chemosensitizer.

Although both GSH and GSSG increased after DMF-containing treatments, the GSH/GSSG ratio remained statistically unchanged, even after log-ratio transformation (Table S3). Because this ratio is a derived variable influenced by the variability of both metabolites, parallel increases in GSH and GSSG may result in only modest ratio changes and limited statistical power to detect treatment-related differences. Our hypothesis is that, as an electrophilic species, DMF may induce a parallel elevation of GSH and GSSG within the measurable glutathione pool, potentially reflecting increased glutathione turnover, adaptive antioxidant buffering, enhanced recycling, or redistribution between soluble and protein-bound thiol species [40,42]. A limitation of this study is that a more comprehensive characterization of glutathione-dependent systems and redox-related enzymatic parameters was not performed. Future studies should evaluate whether DMF-DCT modulates GSH biosynthesis, GSSG-to-GSH recycling, glutathione reductase/peroxidase activity, NADPH availability, and other antioxidant systems involved in the prostate cancer redox response.

Mitochondria play a fundamental role in cell death [43,44], and our results indicate that LNCaP cells display a mitochondrial stress-associated, caspase-linked apoptotic response. DMF and DCT induced early apoptosis in tumor cells, as supported by caspase activation analysis. Furthermore, DMF–DCT promoted cell death through the activation of caspases 3 and 8 in LNCaP cells, consistent with previous reports showing the therapeutic relevance of DMF-induced apoptotic effects [45,46]. However, no caspase-9 activation was detected, indicating that the canonical intrinsic apoptotic pathway was not confirmed under our experimental conditions. In contrast, PC-3 cells did not display this apoptotic profile, which may be related to their intrinsic chemoresistant phenotype. Although DMF has been reported to induce apoptosis, additional cell death pathways may contribute to the reduced viability observed in PC-3 cells, including necroptosis- or autophagy-associated mechanisms [46,47,48,49]. This response was accompanied by lower ROS levels, particularly under the combined DMF-DCT treatment. However, because mitophagy, necroptosis, and mitochondrial membrane integrity were not directly assessed, this interpretation remains speculative and requires further validation.

Oxidative energy metabolism depends on the quantity and quality of mitochondrial function [50]. Therefore, we performed a respirometry assessment to assess mitochondrial function. Although treatments did not induce any significant change in oxygen consumption rate in PC-3 cells, LNCaP cells were affected by DMF-DCT combination in concordance with higher oxidative stress and diminished mitochondrial energy demand (as high maximum oxygen consumption and non-mitochondrial respiration) [51,52]. LNCaP cells also showed an increasing trend in proton leak, which may be linked to damage to the inner mitochondrial membrane [51], and non-mitochondrial respiration typically increases with cellular stressors such as ROS. Since proton leak increase may be linked to damage to the inner mitochondrial membrane [51], and non-mitochondrial respiration typically increases with cellular stressors such as ROS [52], LNCaP cells under the DMF-DCT combination may exhibit a specific pattern of mitochondrial damage. PC-3 results must be confirmed to determine whether other cell death mechanisms are involved.

In this context, the divergent cell death responses may be partially explained by intrinsic molecular differences between LNCaP and PC-3 cells. LNCaP cells retain functional/wild-type p53, whereas PC-3 cells are commonly described as p53-null or p53-deficient [53,54]. In prostate cancer models, p53 functional status has been associated with differential sensitivity to therapeutic agents and with the regulation of apoptotic competence [53]. Therefore, this distinction may help contextualize the stronger apoptotic phenotype observed in LNCaP cells and the more adaptive metabolic–redox response observed in PC-3 cells after DMF–DCT treatment. In contrast, PTEN status should be interpreted with caution, as both LNCaP and PC-3 cells are generally considered PTEN-deficient models [55]. Therefore, PTEN loss may provide a shared PI3K/AKT-related pro-survival and metabolically plastic background rather than a simple factor discriminating the two cell lines [56]. Accordingly, the differential response to DMF–DCT likely reflects the interaction between this shared PTEN-deficient context and additional cell-line-specific determinants, particularly p53 status. Although these pathways were not directly interrogated in the present study, functional approaches and their consideration provide biologically plausible framework to contextualize the phenotype-dependent responses observed here.

Our findings collectively support that DMF functions as a pleiotropic modulator of metabolic and mitochondrial responses associated with docetaxel treatment, with concomitant changes in redox-associated markers. These effects appear to affect cellular stress responses relevant for tumor cell survival through phenotype-dependent mechanisms. This differential response—associated with sustained ROS levels and mitochondrial stress in LNCaP cells, and with reduced mitochondrial mass, lower ROS levels, and changes in glutathione-associated parameters in PC-3 cells—helps contextualize the observed combinatorial effects and reinforces the notion that effective therapeutic strategies may leverage distinct metabolic and mitochondrial vulnerabilities within diverse tumor environments.

## 4. Materials and Methods

### 4.1. Cell Culture Conditions

The human cell lines RWPE-1 (ATCC^®^ CRL-11609), LNCaP (ATCC^®^ CRL-1740), and PC-3 (ATCC^®^ CRL-1435) were obtained from the American Type Culture Collection (ATCC, Manassas, VA, USA). LNCaP and PC3 cell lines were cultured in RPMI 1640 medium (Gibco-Thermo Fisher Scientific, Gaithersburg, MD, USA), supplemented with 3% fetal bovine serum (FBS) (Gibco, Grand Island, NY, USA) and 1% antibiotic cocktail penicillin/streptomycin (Sigma-Aldrich, St. Louis, MO, USA); RWPE-1 was cultured in serum-free keratinocyte basal medium (K-SFM) 1X supplemented with bovine pituitary extract (BPE) and human recombinant epidermal growth factor (rhEGF); and 1% antibiotic cocktail of penicillin/streptomycin was incubated with 5% CO_2_ at 37 °C and humidity ≥95%. They were grown to approximately 80% confluence [57,58,59].

### 4.2. Treatments

For treatments, dimethyl fumarate 97% (242926, Sigma-Aldrich, St. Louis, MO, USA) was used as a 100 mM stock solution in DMSO (Sigma-Aldrich, St. Louis, MO, USA). From this stock, a working solution of 100 μM DMF was prepared in RPMI simple media. Also, serial dilutions were prepared for each final tested DMF concentration at sterile conditions. Furthermore, Taxotere ^®^ docetaxel as a 20 mg/mL vial, (Sanofi-Aventis Deutschland GmbH, Frankfurt, Germany) was used as a 100 uM DCT working solution prepared using RPMI simple media for dilutions. Equally, serial dilutions were prepared for each final tested DCT concentration at sterile conditions.

### 4.3. Cell Viability Assay

The viability of the PC-3, LNCaP, and RWPE-1 cells under treatment conditions was assessed using crystal violet staining, and baseline IC50 (mean inhibitory concentration) values were determined for each drug. Initially, PC-3, LNCaP, and RWPE-1 cells were seeded in 96-well microplates at a density of 5 × 10^4^ cells/well and allowed to adhere and grow for 24 h [9]. After this time, cells were incubated in media containing DMF and DCT at concentrations of 0, 1, 10, 20, 50, 150, 200, 300 µM and 0, 0.03, 0.06, 0.1, 1, 1.5, 3 µM, respectively, for 24, 48, or 72 h. At the end of the incubation periods, media was extracted for metabolic measurements (glucose and lactate levels). Cells adhered to the microplate were washed with phosphate-buffered saline (PBS 1X) (Sigma-Aldrich, St. Louis, MO, USA) and fixed to the microplate by adding 100 µL of 4% paraformaldehyde solution to each well for 30 min at room temperature. The CV assay was performed as previously described [60]. Absorbance was read at 570 nm. Crystal violet staining was quantified on a BioTek^®^ Epoch 2 microplate spectrophotometer using Gen5^®^ Microplate Reader software (V3.05.11 from BioTek Instruments, Inc., Winooski, VT, USA). Dose–response curves were performed, and each experiment was performed in triplicate.

Absorbance values were normalized to the untreated control cultures, which were defined as 100%. Normalized data were then plotted as dose–response curves. IC50 values were estimated for each cell line by nonlinear regression analysis using the Hill slope model in GraphPad Prism Software version 9. Only curves showing adequate goodness of fit (R^2^ > 0.95) were considered for IC50 calculation.

### 4.4. Cytotoxicity Assay

The effects of DMF and DCT on cellular metabolic activity were determined using the MTT (3-(4,5-dimethylthiazol-2-yl)-2,5-diphenyltetrazolium bromide) assay, as a measure of cytotoxicity [61,62]. RWPE-1, LNCaP, and PC-3 cells were seeded in 96-well microplates at a density of 5 × 10^4^ cells/well and allowed to attach for 24 h. Cells were then exposed to media containing the previously determined IC50 concentrations of DMF and DCT. The MTT assay was performed as previously described [63]. After MTT removal, formazan crystals deposited at the bottom of each well were dissolved in 100 μL of DMSO. Absorbance was measured using a BioTek^®^ Epoch 2 microplate spectrophotometer and Gen5^®^ Microplate Reader software (v3.05.11; BioTek Instruments, Inc.). Dose–response curves were generated, and all experiments were performed in triplicate.

### 4.5. Combination Analysis

To characterize potential interactions between DMF and DCT, dose–response relationships were established across treatments. For this purpose, IC50 values were calculated, and data were obtained using the previously described crystal violet test; these IC50 values were determined at the optimal exposure times based on the observed effects at each dose and exposure time. Dose-combination tests were performed between DMF and DCT at different concentration ratios (1:1, 1:2, and 2:1). Possible synergistic, additive, or antagonistic responses were estimated by constructing isobolograms as described by Tallarida [27,28]. In addition, further analysis was performed using CalcuSyn software (version 1.0) to quantify combination and dose-reduction indexes for DMF-DCT cell viability data.

For combination assays, we performed experiments as described in our previous work [30]. To evaluate combinatorial effects, cell viability was assessed by crystal violet staining using IC50-derived combination schemes. Briefly, fixed 1:1, 1:2, and 2:1 combination ratios were used to generate five-point dose–response curves by applying either decremental or incremental scaling of the IC50 values. Specifically, for conditions based on 1/2 × IC50, serial dilutions were prepared by stepwise reductions from 1/2 × IC50 for each agent, according to the selected ratio. In contrast, for conditions based on 1.5 × or 2 × IC50, concentrations were increased stepwise up to the indicated multiples of the IC50.

Cells were treated with the corresponding combination ratios prepared in culture medium, with a final DMSO concentration ≤0.1% (*v*/*v*), for a total incubation period of 48 h. No pretreatment protocol was used; DMF and DCT were administered simultaneously. After the first 24 h of exposure, the treatment-containing medium was replaced with freshly prepared medium containing the same DMF–DCT combination, and cells were then incubated for an additional 24 h to complete the 48-h treatment period. After treatment, cell viability was assessed by crystal violet staining and normalized to untreated controls. Dose–response curves were constructed and analyzed to determine the most effective combination ratio based on potency shifts and IC50 displacement for each cell line.

### 4.6. Intracellular Reactive Oxygen Species Determination

Intracellular levels of ROS were determined with the use of the fluorescent probe 5-(y-6)-chloromethyl-2’7’-dichlorodihydroflourescein diacetate (CM-H2DCFDA) (C6827 Invitrogen™, Eugene, OR, USA) [64]. The increase in fluorescence due to oxidation of the probe was detected by flow cytometry. At the end of the 48-h period, cells were detached with TrypLE^®^ Express 1X and resuspended 8 × 10^5^ in 500 µL a solution with CM-H2DCFDA 2.75 µM in RPMI 1640 medium (Gibco, Grand Island, NY, USA) without supplementation, for 45 min in the dark at 37 °C. One group of cells was incubated without probe as a negative control of the assay. Then, they were centrifuged, the probe-containing medium was removed, and the cells were resuspended in 400 µL of 1X PBS. Data acquisition was made using the Cytek™ Northern Lights flow cytometer (Cytek Biosciences, Fremont, CA, USA), and SpectroFlo^®^ Sofware (version 3.0.1). Fluorescence is measured at a wavelength Ex/Em: ∼492–495/517–527 nm [65]. The acquired data were processed in FlowJo™ V.10.8.1 (BD Life Sciences, Ashland, OR, USA) software to determine the relative fluorescence intensity (RFI) per cell. ROS levels are expressed as RFI normalized data from control.

### 4.7. Relative Mitochondrial Mass Determination

Relative mitochondrial mass was determined by staining with MitoTracker Green following manufacturer instructions. For this purpose, after the incubation period with DMF and DCT, cells were detached using TrypLE^®^ Express 1X and resuspended 8 × 10^5^ in 500 µL of a solution (150 nM) of MitoTracker Green FM probe (M7514 Invitrogen™, Eugene, OR, USA) pre-warmed to 37 °C. Incubated at 37 °C in 95% air and 5% CO_2_ atmosphere for 45 min. Then, the cells were centrifuged, the medium containing the probe was removed, and the cells were resuspended in 0.4 mL of 1X PBS. Data were acquired using the Cytek™ Northern Lights flow cytometer (Cytek Biosciences, Fremont, CA, USA) and SpectroFlo^®^ software (version 3.0.1). Relative mitochondrial mass is expressed as RFI normalized data. It is calculated by dividing the fluorescence intensity of each determination by that obtained for the control (untreated cells) in each experiment. Fluorescence is measured at a wavelength Ex/Em: ∼492–495/517–527 nm [65]. The acquired data were processed in FlowJo™ V.10.8.1 (BD Life Sciences, Ashland, OR, US) software to determine the relative fluorescence intensity per cell and, thus, the relative mitochondrial mass [26].

### 4.8. GSH/GSSG Ratio Determination

The measure of GSH/GSSG ratio was obtained using the reduced (GSH) and oxidized (GSSG) glutathione quantification kit (38185 Sigma-Aldrich, St. Louis, MO, USA). The kit is based on the colorimetric reaction of 5,5’-dithiobis (2-nitrobenzoic acid) and absorbance measurement at 412 nm. Cells (1 × 10^7^) were collected by centrifugation at 200× *g* 10 min at 4 °C, the supernatant was discarded, and the cells were washed with PBS 1X and centrifuged once more at 200× *g* 10 min at 4 °C, the supernatant was discarded, 80 μL of HCl was added, the cells were lysed by freezing and thawing periods at least twice. Next, 20 μL of 5% SSA was added and centrifuged at 8000× *g* 10 min. Finally, the supernatant was transferred to a new tube by adding ddH_2_O to reduce the SSA concentration of the assay to 0.5%, following the manufacturer’s guidelines. Absorbance reading was performed using an BioTek^®^ Epoch 2 microplate spectrophotometer at 412 nm (endpoint reading), using Gen5^®^ Microplate Reader software (V3.05.11 from BioTek Instruments, Inc., USA). Results were expressed as μmol/μL. GSH and GSSG concentrations were calculated from standard curves generated according to the kit protocol. Because all samples were prepared from the same number of cells and processed using identical extraction and dilution volumes, GSH and GSSG levels were expressed as glutathione content in standardized extracts obtained from 1 × 10^7^ cells, rather than as values normalized to an arbitrary lysate volume. The GSH/GSSG ratio was calculated as a complementary index of cellular redox status.

### 4.9. Glucose Intake and Lactate Production

Basal measurements of glucose intake and lactate production was measured at 24, 48, and 72 h Samples of the culture medium were collected for each time and individual treatment, from untreated and DMF- and DCT-treated cells and deproteinized using the Deproteinization TCA kit (ab204708 abcam) and Halt™ protease inhibition cocktail, EDTA-free (Thermo Scientific™). Samples were stored at −20 °C, until use.

The levels of initial glucose and remaining glucose in the culture medium to obtain the cellular consumption of glucose were determined with the SIGMA GAGO20 glucose assay kit (Sigma-Aldrich, St. Louis, MO, USA), following the manufacturer’s instructions and miniaturizing the assay in a 96-well microplate [66]. The absorbance of each well was homogenized and read at 540 nm in a BioTek^®^ Epoch 2 microplate spectrophotometer using Gen5^®^ Microplate Reader software (V3.05.11 from BioTek Instruments, Inc., USA). The results are expressed in µg/µL of glucose; the data were normalized considering the concentration obtained from untreated cultures.

The lactate level at the end of the 48-h period was determined using the SIGMA MAK064 lactate assay kit (Sigma-Aldrich, St. Louis, MO, USA), following the manufacturer’s instructions [67,68]. For each one, 50 µL of sample + 50 µL of the kit’s master mix were taken in each well. First, the samples were homogenized using a horizontal orbital shaker; then, the microplate was incubated for 30 min at room temperature in the dark. After this time, the absorbance was read at 570 nm in a BioTek^®^ Epoch 2 microplate spectrophotometer using Gen5^®^ Microplate Reader software (V3.05.11 from BioTek Instruments, Inc., USA). The results are expressed as nmoles/µL of lactate; the data were normalized considering the concentration obtained from untreated cultures.

### 4.10. Flow Cytometric Analysis of Apoptosis

In PC-3 and LNCaP prostate cancer cell cultures, the distribution of cells at different stages of apoptosis was determined by detecting the translocation of phosphatidylserine (PS) to the outer surface of the membrane, where it facilitates the binding of Annexin-V to PS.

A marker of dead cells was also used as an indicator of cell membrane structural integrity, such as the intercalation of 7-AminoActinomycin D (7-AAD) to the double-stranded DNA of damaged and dead cells, using the kit Muse ^®^ Annexin V & Dead Cell (MCH 100,105 Luminex Corp., Austin, TX, USA), following the manufacturer’s recommendations. After the exposure time of the assay (48 h) with each of the treatments, PC3 and LNCaP cells were detached with TrypLE^®^ Express 1X; when the detachment was achieved, the action of trypsin was stopped with SFB and the cell button was resuspended by centrifugation at 1200 RPM. Finally, the cell button was resuspended in a medium with a serum to a final concentration of 2 × 10^5^ cells/mL.

Staining of cells was performed from the kit previously tempered at 25 °C, and in 1.5 mL conical tubes, the addition of 100 μL of cells in suspension resuspended in 1X PBS for each tube, along with the addition of 100 μL of the kit reagent to each tube. The resulting mixture was homogenized. Each tube with the sample was incubated for 20 min at room temperature, protected from light. The percentage of apoptotic cells was analyzed by flow cytometry using the Muse™ Cell Analyzer system (Millipore Corp., Hayward, CA, USA) and expressed as the percentage of apoptotic cells and standard error of mean. Cells without treatment were used as a negative control.

### 4.11. Caspase 3, 6 and 9 Determinations

To know the mechanisms related to the induction of death in cells treated with DMF and DCT, the detection of caspases was performed using cultures and treatments in 96-well black microplates with a clear bottom, in which 2 × 10^4^ cells/well were seeded and treated with the IC50 of DMF and DCT at the best exposure time previously determined. For PC-3 and LNCaP cell line, there was a control group (untreated) and a group treated with DMF and DCT. After the treatments, caspases were evaluated using the Caspase 3, Caspase 8 & Caspase 9 Multiplex Activity Assay Kit Fluorometric Kit (ab219915, Abcam, CA, UK), following the manufacturer’s recommendations.

Into these cells, 100 µL/well of loading solution was added directly onto the microplate after the incubation time without removing the culture medium/treatment; the plate was incubated again at room temperature for 1 h, protected from light. Fluorescence values were read in the CLARIOstar^®^ fluorescence microplate reader (BMG Labtech, OG, Germany) at wavelengths of Ex/Em = 535/620 nm (red) for Caspase 3, Ex/Em = 490/525 nm (green) for Caspase 8 and Ex/Em = 370/450 nm (blue) for Caspase 9. The results are expressed as relative fluorescence units (RFU).

### 4.12. Real-Time Reverse Transcription Polymerase Chain Reaction Analysis (RT-qPCR)

Gene expression levels of transcripts related to redox regulation and glycolytic metabolism were assessed by real-time quantitative reverse transcription PCR (RT-qPCR) focusing on *SOD2* and *LDHA*, respectively.

Total RNA was isolated from cell lines at the end of each experiment using the PureLink^®^ RNA Mini Kit (12183025, Invitrogen™, Carlsbad, CA, USA) according to the manufacturer’s instructions. RNA purity and concentration were measured using a NanoDrop^®^ Spectrophotometer (ND-1000, Wilmington, DE, USA). cDNA synthesis was performed from 1 µg of total RNA using the High Capacity cDNA Reverse Transcription Kit (Thermo Fisher, London, UK) in a T100™ thermal cycler (Bio-Rad, Hercules, CA, USA) following the manufacturer’s protocol. The reverse transcription conditions were 25 °C for 10 min, 37 °C for 2 h, 85 °C for 5 min, and 4 °C for hold. Expression assays were performed with TaqMan ^®^ probes (Thermo Fisher): SOD2 FAM-MGB probe (Hs00167309_m1) and LDHA FAM-MGB probe (ID: Hs01378790_g1). The 18S VIC-MGB (ID:Hs03003631_g1) was used as the endogenous control. A total of 100 ng of cDNA was amplified in a final reaction volume of 20 µL containing TaqMan^®^ Universal PCR Master Mix (Applied Biosystems, Warrington, UK). Quantification was performed on the Cobas^®^ Z-480 system (Roche Diagnostics, Rotkreuz, Switzerland) and LightCycler^®^ Systems Training software (Expert Level) version LCS480 1.5.1.62 SP3, UDF 2.1.0.26 (Roche Diagnostics) using the relative quantification (RQ) method [69]. Each sample was analyzed in triplicate.

The qPCR cycling conditions were as follows: polymerase activation at 95 °C for 10 min, followed by 40 cycles of denaturation at 95 °C for 15 s and annealing/extension at 60 °C for 60 s. Relative expression levels were visualized as heatmaps with accompanying dendrograms using the pheatmap (version 1.0.13) and ggplot2 (version 4.0.1) packages in R.

### 4.13. Respirometry Analysis

Cellular respiration in RWPE-1, LNCaP, and PC-3 cells was evaluated under basal conditions using a high-resolution Oxygraph-2k respirometer (Oroboros Instruments, Innsbruck, Austria) at 37 °C with continuous gentle stirring. Both untreated and treated cells (DMF, DCT, and DMF-DCT) were analyzed.

Mitochondrial function was assessed through sequential addition of oligomycin (2.2 μM), FCCP (0.75 μM for PC-3 and 0.5 μM for LNCaP), and the inhibitors antimycin A (0.5 μM) plus rotenone (0.5 μM). Oxygen consumption rates were recorded to determine basal respiration, proton leak, maximal respiratory capacity, and non-mitochondrial respiration. Data were normalized per cell (pmol O_2_·s^−1^·cell^−1^) and expressed as mean ± SEM from three independent experiments.

### 4.14. Statistical Analysis

The number of biological replicates for each experiment is indicated in the corresponding figure legends. Technical replicates were included according to the requirements of each assay when applicable. Data are presented as mean ± SEM.

Statistical comparisons between groups were performed using one-way ANOVA followed by Bonferroni post hoc tests. When assumptions of normality or homogeneity of variance were not satisfied, non-parametric analyses (Kruskal–Wallis followed by Mann–Whitney multiple comparisons) were applied. Dose–response relationships and drug interaction analyses were assessed by nonlinear regression using GraphPad Prism software version 11.0.2 (92) (GraphPad Software, Boston, MA, USA). Combination index analyses were performed using CalcuSyn software. Statistical significance was established at *p* < 0.05.

## 5. Conclusions

In summary, our study shows that dimethyl fumarate modulates docetaxel responses in prostate cancer cells in a phenotype-dependent manner. Rather than acting as a uniform docetaxel chemosensitizer, DMF–DCT efficacy was shaped by drug concentration, combination ratio, and the intrinsic biological features of each cell model. The most favorable pharmacological interaction windows were observed in balanced or DCT-enriched regimens, particularly in PC-3 cells, where the combination produced a relevant docetaxel dose-sparing effect. These findings support a context-dependent potentiation of docetaxel response rather than generalized chemosensitization.

DMF–DCT exposed divergent phenotype-dependent vulnerabilities: a mitochondrial stress-associated, caspase-linked apoptotic response in LNCaP cells, and a distinct adaptive mitochondrial response in PC-3 cells with limited apoptotic engagement and changes in glutathione-associated parameters. Crucially, a potentially advantageous therapeutic window for this approach is suggested by the low toxicity seen in RWPE-1 cells.

Overall, DMF emerges as a context-dependent metabolic modulator of docetaxel response, with associated changes in mitochondrial function, ROS levels, *SOD2* expression, and the measurable glutathione pool. These findings provide a rationale for further evaluation of fumarate-based strategies to modulate taxane responses in prostate cancer. Future studies using advanced preclinical models will be important to define the translational relevance of this combination and to determine whether it may help reduce docetaxel-associated toxicity while maintaining antitumor efficacy.

## Figures and Tables

**Table 1:** Dose reduction index (DRI) analysis of DMF–DCT combinations in PC-3 cells. Dose reduction indices (DRI) were calculated for DCT and DMF across fixed-ratio combinations (1:1, 2:1, and 1:2) using the Chou–Talalay method implemented in CompuSyn software (version 1.0). When compared to single-agent treatment at equivalent effect levels, the DRI shows the fold reduction in drug dose that can be achieved in combination. A favorable dose reduction is indicated by values greater than 1. The results, which correspond to increasing doses of DMF and DCT, are shown as mean ± standard deviation (SD) from three concentration levels per ratio. These findings demonstrate a preferential dose-sparing effect for DCT, especially in situations where DCT predominates.

DCT:DMF Combination Ratio	DCT (µM)	DMF (µM)	DCT DRI	DMF DRI
1:1	0.01	47.50	42.44 ± 24.53	13.79 ± 2.45
	0.02	71.25		
	0.02	95.00		
2:1	0.01	95.00	52.67 ± 21.99	8.00 ± 2.26
	0.02	142.50		
	0.02	190.00		
1:2	0.02	47.50	22.03 ± 11.63	14.20 ± 2.54
	0.03	71.25		
	0.04	95.00		

## Data Availability

The raw data supporting the conclusions of this article will be made available by the authors on request.

## Supplementary Materials

The following supporting information can be downloaded at: https://www.mdpi.com/article/10.3390/ijms27146209/s1.
