# Supplementary material for: Metabolic Modulation by Dimethyl Fumarate Alters Docetaxel Responses in Prostate Cancer Cells
Source: Int J Mol Sci. 2026 Jul 11;27(14):6209. doi: 10.3390/ijms27146209 (PMC13411018; doi:10.3390/ijms27146209)
Supplement: Supplementary file 1 [file ijms-27-06209-s001.zip › ijms-4292058-supplementary/Figure S1.pdf]

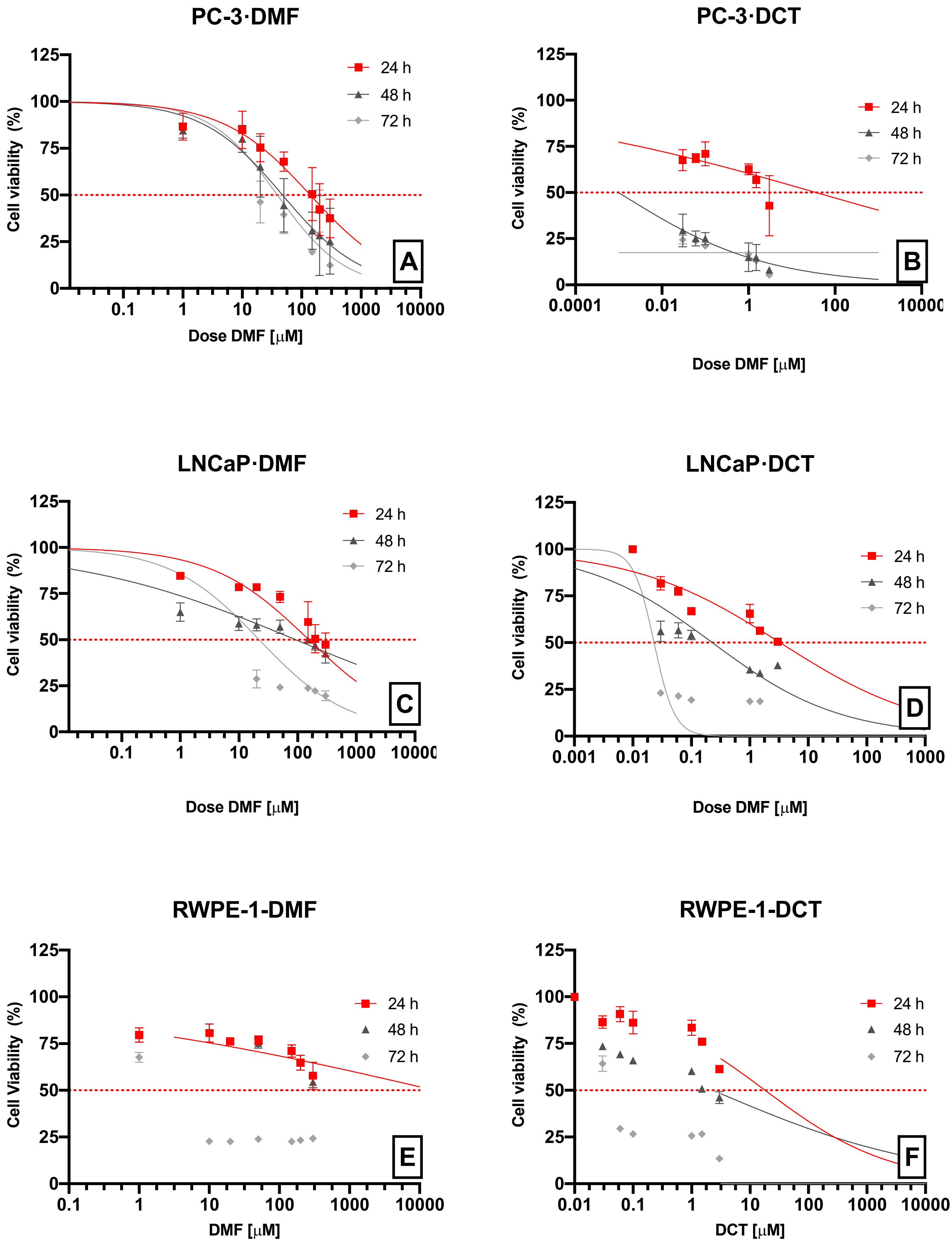

Figure S1. Time-dependent effects of DMF and DCT on cell viability in prostate cancer cell lines. Cell viability dose–response curves for dimethyl fumarate (DMF) and docetaxel (DCT) were generated in prostate cancer cell lines at different time points. (A, B) PC-3 cells, (C, D) LNCaP cells, and (E, F) RWPE-1 cells were treated with increasing individual concentrations of DMF or DCT for 24, 48, and 72 h, and cell viability was assessed to determine time-dependent pharmacological responses. Nonlinear regression models were applied to fit dose–response curves and estimate IC50 values for each compound. Data were collected in triplicate across three independent experiments and are expressed as mean ± standard error of the mean (SEM). All concentrations are expressed in μM.
