# Supplementary material for: Metabolic Modulation by Dimethyl Fumarate Alters Docetaxel Responses in Prostate Cancer Cells
Source: Int J Mol Sci. 2026 Jul 11;27(14):6209. doi: 10.3390/ijms27146209 (PMC13411018; doi:10.3390/ijms27146209)
Supplement: Supplementary file 1 [file ijms-27-06209-s001.zip › ijms-4292058-supplementary/Figure S2.pdf]

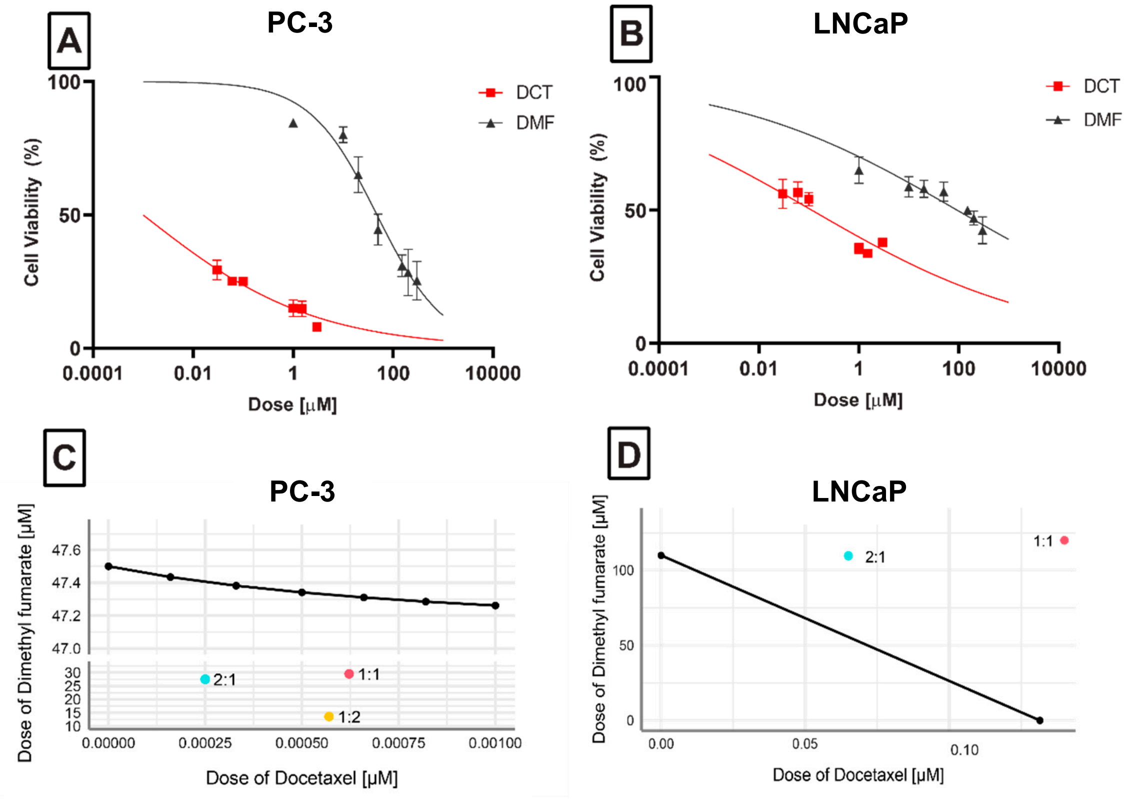

Figure S2. Effects of DMF-DCT combinations yields phenotype and dose-dependent pharmacological interactions in prostate cancer cells. Relative potency curves and isoboles are depicted. **(A)** Cell viability curves and non-linear model for calculation of potency ratios for DMF and DCT administered alone on PC3 cells. **(B)** Cell viability curves and non-linear model for calculation of potency ratios for DMF and DCT administered alone on LNCaP cells. **(C)** Curve-fitted isobologram obtained from the combination analysis performed on PC-3 cells treated with IC50 of DMF and DCT at 48 h showed a global synergistic effect at 1:1, 1:2, and 2:1 DMF-DCT ratios. **(D)** Linear-fitted isobologram obtained from the combination analysis performed on LNCaP cells treated with IC50 of DMF and DCT at 48 h showed global antagonism at 1:1, 1:2, and 2:1 DMF-DCT ratios. Values were plotted in μM.
