# Supplementary material for: Metabolic Modulation by Dimethyl Fumarate Alters Docetaxel Responses in Prostate Cancer Cells
Source: Int J Mol Sci. 2026 Jul 11;27(14):6209. doi: 10.3390/ijms27146209 (PMC13411018; doi:10.3390/ijms27146209)
Supplement: Supplementary file 1 [file ijms-27-06209-s001.zip › ijms-4292058-supplementary/Figure S3.pdf]

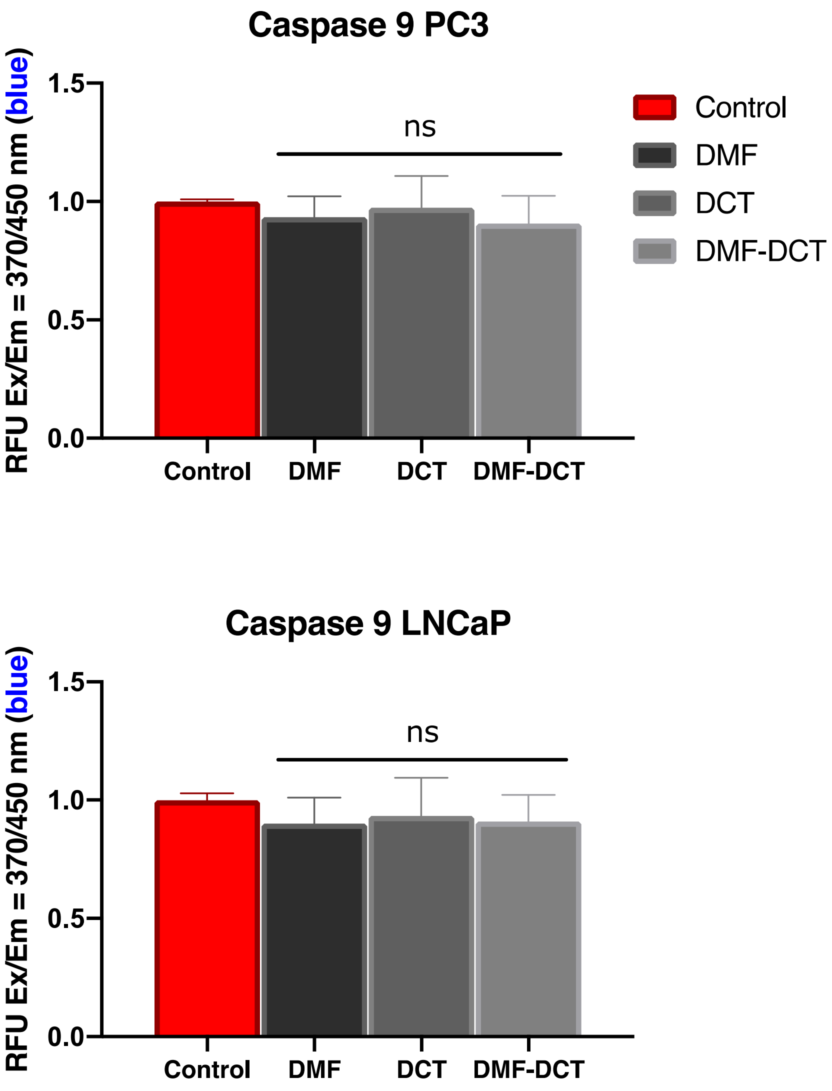

Figure S3. Caspase-9 activity in PC-3 and LNCaP cells after treatment. Caspase-9 activity was measured in PC-3 and LNCaP cells after 48 h of treatment with vehicle/control, DMF, DCT, or the DMF–DCT combination. Fluorescence values are presented as relative fluorescence units or normalized values according to the corresponding untreated control. Data are shown as mean ± SEM from three independent biological experiments (n = 3).
