# Supplementary material for: Metabolic Modulation by Dimethyl Fumarate Alters Docetaxel Responses in Prostate Cancer Cells
Source: Int J Mol Sci. 2026 Jul 11;27(14):6209. doi: 10.3390/ijms27146209 (PMC13411018; doi:10.3390/ijms27146209)
Supplement: Supplementary file 1 [file ijms-27-06209-s001.zip › ijms-4292058-supplementary/Table S1_Final.pdf]

Table S1: Half-maximal inhibitory concentration (IC50) values for dimethyl fumarate (DMF) and docetaxel (DCT) in RWPE-1, LNCaP, and PC-3 cell lines at 24, 48, and 72 h. Values are expressed in μM and were calculated from dose–response curves based on cell viability assays. Scientific notation is used for very low concentrations. This table highlights time-dependent and cell line–specific differences in drug sensitivity.

	**Cell lines**								
	**RWPE-1**			**LNCaP**			**PC-3**		
**Treatment/time**	24 h	48 h	72 h	24 h	48 h	72 h	24 h	48 h	72 h
**DMF (μM)**	4848	2882	2,1	306,4	109,8	0.5	139,2	47,5	14,1
**DCT (μM)**	26,1	2,3	0,02	3,7	0,1	$1.0x10^{-8}$	16,2	0,01	0,001
