# Supplementary material for: Metabolic Modulation by Dimethyl Fumarate Alters Docetaxel Responses in Prostate Cancer Cells
Source: Int J Mol Sci. 2026 Jul 11;27(14):6209. doi: 10.3390/ijms27146209 (PMC13411018; doi:10.3390/ijms27146209)
Supplement: Supplementary file 1 [file ijms-27-06209-s001.zip › ijms-4292058-supplementary/Table S2.pdf]

Table S2: Combined data from cell viability responses in LNCaP and PC-3 cells with combination indexes computed using the Chou-Talalay median effect method. Cells were exposed to fixed concentrations of DCT:DMF in combination ratios for 48 hours. The results of the cell viability tests are reported as the mean ± standard deviation (SD). CI interpretation is based on established criteria. Red colours indicate antagonism, whereas green colours indicate synergy.

Cell line	DCT:DMF combination ratio	DMF (μM)	DCT (μM)	Cell viability (%)	Calculated CI	CI interpretation
LNCaP	1:1	6,86	0,008	65,44 ± 15,16	5,58	strong antagonism
	1:1	13,73	0,016	71,66 ± 29,2	70,46	strong antagonism
	1:1	27,45	0,033	69,83 ± 24,96	79,83	strong antagonism
	1:1	54,90	0,066	79,72 ± 4,00	5039,49	strong antagonism
	1:1	109,80	0,132	68,15 ± 14,90	193,30	strong antagonism
	1:1	164,70	0,197	23,09 ± 13,65	0,01	very strong synergism
	1:1	219,60	0,263	7,83 ± 3,52	$2,9 \times 10^{-5}$	very strong synergism
	2:1	13,73	0,008	71,66 ± 29,2	66,46	strong antagonism
	2:1	27,45	0,016	89,33 ± 19,02	382533,63	strong antagonism
	2:1	54,90	0,033	75,39 ± 17,75	940,59	strong antagonism
	2:1	109,80	0,066	66,60 ± 12,29	112,67	strong antagonism
	2:1	219,60	0,132	76,94 ± 19,99	6627,92	strong antagonism
	2:1	439,20	0,263	7,83 ± 3,52	$2,9 \times 10^{-5}$	very strong synergism
	1:2	6,86	0,016	65,44 ± 15,16	6,67	strong antagonism
	1:2	13,73	0,033	71,46 ± 24,75	73,81	strong antagonism
	1:2	27,45	0,066	66,76 ± 31,50	38,11	strong antagonism
	1:2	54,90	0,132	52,60 ± 14,37	2,56	antagonism
	1:2	109,80	0,263	61,37 ± 9,66	37,94	strong antagonism
	1:2	164,70	0,395	23,08 ± 13,65	0,01	very strong synergism
	1:2	219,60	0,526	7,83 ± 3,52	$5,8 \times 10^{-5}$	very strong synergism
PC-3	1:1	2,97	0,001	89,78 ± 7,49	575,90	strong antagonism
	1:1	5,94	0,001	71,21 ± 34,64	15,81	strong antagonism
	1:1	11,88	0,003	77,0 ± 10,36	87,50	strong antagonism
	1:1	23,75	0,005	73,12 ± 8,38	87,10	strong antagonism
	1:1	47,50	0,010	18,56 ± 1,59	0,11	strong synergism
	1:1	71,25	0,015	16,85 ± 3,47	0,12	strong synergism
	1:1	95,00	0,020	12,45 ± 2,08	0,08	very strong synergism
	2:1	5,94	0,001	89,78 ± 7,49	579,51	strong antagonism
	2:1	11,88	0,001	96,89 ± 8,85	86052,75	strong antagonism
	2:1	23,75	0,003	82,73 ± 2,56	298,32	strong antagonism
	2:1	47,50	0,005	63,49 ± 3,38	21,15	strong antagonism
	2:1	95,00	0,010	15,53 ± 4,42	0,12	strong synergism
	2:1	142,50	0,015	16,85 ± 3,47	0,21	strong synergism
	2:1	190,00	0,020	12,45 ± 2,08	0,14	strong synergism
	1:2	2,97	0,001	89,78 ± 7,49	1148,20	strong antagonism
	1:2	5,94	0,003	95,21 ± 2,41	37019,39	strong antagonism
	1:2	11,88	0,005	73,92 ± 3,15	97,97	strong antagonism
	1:2	23,75	0,010	68,41 ± 8,27	79,03	strong antagonism
	1:2	47,50	0,020	17,86 ± 3,44	0,13	strong synergism
	1:2	71,25	0,030	16,85 ± 3,47	0,16	strong synergism
	1:2	95,00	0,040	12,45 ± 2,08	0,09	very strong synergism
