# Supplementary material for: Metabolic Modulation by Dimethyl Fumarate Alters Docetaxel Responses in Prostate Cancer Cells
Source: Int J Mol Sci. 2026 Jul 11;27(14):6209. doi: 10.3390/ijms27146209 (PMC13411018; doi:10.3390/ijms27146209)
Supplement: Supplementary file 1 [file ijms-27-06209-s001.zip › ijms-4292058-supplementary/Table S3_Final.pdf]

Table S3. Log-transformed GSH/GSSG ratio analysis and geometric fold-change estimates in PC-3 and LNCaP cells after DMF, DCT, and DMF–DCT treatments. Data shows log10-transformed GSH/GSSG ratio and geometric fold changes relative to untreated controls in PC-3 and LNCaP cells after treatment with dimethyl fumarate (DMF), docetaxel (DCT), or their combination (DMF–DCT). Log transformation was used as a complementary approach for ratio-based data. In both cell lines, all 95% confidence intervals crossed unity, indicating no statistically robust directional shift in the soluble GSH/GSSG redox balance.

Cell line	Treatment	Mean log10(GSH/GSSG)	Geometric FC vs control	IC95%
PC-3	DMF	1.994	0.694	0.17–2.88
PC-3	DCT	1.880	0.534	0.098–2.91
PC-3	DMF-DCT	2.004	0.709	0.12–4.15
LNCaP	DMF	1.583	1.197	0.004–338.14
LNCaP	DCT	1.591	1.219	0.005–329.39
LNCaP	DMF-DCT	1.841	2.168	0.035–133.16
